# CD14 in Salt‐Sensitive Hypertension‐Related Cognitive Impairments

**DOI:** 10.1002/alz.087018

**Published:** 2025-01-03

**Authors:** Chengyun Tang, Emily Burns, Jane J. Border, David Bunn, Cameron Cantwell, Andrew Gregory, Claire Johnson, Yanbin Dong, Richard J. Roman, David L Mattson, Fan Fan

**Affiliations:** ^1^ Augusta University, Augusta, GA USA; ^2^ University of Mississippi Medical Center, Jackson, MS USA

## Abstract

**Background:**

Hypertension is a leading risk factor for the development of Alzheimer’s disease and Alzheimer’s disease‐related dementia (AD/ADRD), which is closely linked with cerebral vascular inflammation and dysfunction. We previously found that high‐salt‐treated Dahl Salt‐Sensitive (SS) rats displayed blood‐brain barrier (BBB) leakage, astrocyte activation, neurodegeneration, and cognitive impairments. CD14 functions in the Toll‐like receptor 4 (TLR4) complex to initiate proinflammatory signaling events in response to LPS. CD14 levels were elevated in the brains of human and animal AD/ADRD models. This study aims to explore the vascular contribution of CD14 to AD/ADRD.

**Method:**

The expression of *Cd14* was assessed through RT‐PCR in primary cerebral vascular smooth muscle cells (VSMCs) isolated from TgF344‐AD rats, and the results were compared to cells from control rats. The myogenic response of the middle cerebral artery (MCA) was compared between SS (SS^CD14+/+^), SS^CD14‐/‐^, and SD rats with and without the induction of hypertension with 4% NaCl diets. BBB function was detected by the leakage of injected Evans blue and fibrinogen. Brain cytokine levels were detected with Bio‐Plex Rat Cytokine 23‐Plex Assay.

**Result:**

*Cd14* emerged as one of the significantly upregulated top genes in high‐salt‐fed SS^CD14+/+^ rats, with a subsequent three‐fold increase observed specifically in cerebral VSMCs of AD compared with control rats. Hypertensive SS^CD14+/+^ rats exhibited an impaired myogenic response of the MCA compared to normotensive SS^CD14+/+^ rats and SD rats fed with both high‐ and low‐salt diets. Notably, the deletion of CD14 demonstrated a remarkable trend in enhancing the myogenic response of the MCA in female SS^CD14‐/‐^ rats. Furthermore, high salt‐fed 24‐week SS^CD14+/+^ rats displayed BBB dysfunction. An augmented pan‐cytokine panel was observed in low salt‐fed 12‐week SS^CD14+/+^ rats, and this effect was magnified in high salt‐fed 24‐week SS^CD14+/+^ rats.

**Conclusion:**

These results collectively point to the potential role of CD14 in influencing vascular and immune responses, suggesting a link between CD14, hypertension, and cerebrovascular pathologies, particularly in the context of AD. Additional investigations are warranted to delve into the underlying mechanisms and ascertain whether CD14 exhibits a sex‐specific role in AD/ADRD.

